# Targeted inhibition of ACLY expression to reverse the resistance of sorafenib in hepatocellular carcinoma

**DOI:** 10.7150/jca.52778

**Published:** 2022-01-04

**Authors:** Hong Sun, Fengchao Wang, Yongqiang Huang, Jin Wang, Lunjun Zhang, Yong Shen, Chao Lin, Pu Guo

**Affiliations:** 1 Department of Clinical Laboratory Science, The First Affiliated Hospital of Bengbu Medical College, Bengbu, China; 2 Department of Nuclear Medicine, The First Affiliated Hospital of Bengbu Medical College, Bengbu, China; 3 The Institute for Advanced Materials and Nano Biomedicine, Tongji University, Shanghai, China

**Keywords:** sorafenib resistance, hepatocellular carcinoma, ACLY, lipids, synthesis, SS-PEI

## Abstract

Resistance to sorafenib has been documented in hepatocellular carcinoma (HCC) patients. We investigated: (i) the correlation between adenosine triphosphate citrate lyase (ACLY) expression and sorafenib resistance in HCC; and (ii) if targeted inhibition could reverse sorafenib resistance. Samples of HCC tissue were obtained from patients and ACLY expression was measured. PET/CT was employed to measure maximum standard unit value (SUV_max_) in HCC patients before and after sorafenib treatment. Using HepG2 cells, we created a sorafenib-resistant cell line. Glucose metabolism and lipid synthesis in HCC cells were tested using ^14^C-glucose. Disulfide-crosslinked polyethylenimine (SS-PEI)-mediated plasmid transfection was carried out, followed by creation of an HCC model in mice. SUV_max_ of HCC lesions was closely related to ACLY expression. Patients with high ACLY expression were not sensitive to sorafenib therapy. Lipid metabolism was more active in sorafenib-resistant HCC cells. ACLY expression was higher in sorafenib-resistant cells and HCC-cell sensitivity to sorafenib increased after ACLY-knockout. The latter reversed sorafenib resistance in HCC cells more significantly under hypoxic conditions. SS-PEI/proline-modified short hairpin-(psh)RNA-ACLY plus sorafenib inhibited the growth of drug-resistant cells significantly. These data suggest that ACLY downregulation can reverse sorafenib resistance, and that SS-PEI can be used to mediate shRNA-ACLY transfection in HCC treatment.

## Introduction

There is a gradually increasing trend in the incidence and mortality of hepatocellular carcinoma (HCC). HCC is the fifth most prevalent cancer worldwide. In China, because of the high incidence of infection by the hepatitis-B virus, ~11 million patients die from hepatocellular cancer each year, accounting for 45% of HCC-related deaths worldwide^1^, ^2^. Surgery is the first-line treatment for HCC, but the recurrence rate is high, especially for patients at middle or advanced stages, who must have other treatments, such as sorafenib^3^.

Sorafenib is an antitumor, multiple-target drug given orally. It inhibits the activity of tyrosine kinase receptors and downstream serine/threonine kinase, thereby inhibiting tumor angiogenesis and promoting tumor-cell apoptosis. Sorafenib can prolong the survival of patients with advanced HCC, and has become the first-line therapy for advanced HCC^4^, ^5^. However, the development of HCC leads to significant drug resistance. Hence, exploring the mechanism of sorafenib resistance and trying to reverse it has become an urgent clinical issue.

In recent years, targeting the metabolism of tumor cells has become an important strategy in the diagnosis and treatment of cancer. In tumor cells, to meet the requirements for lipid skeletons and adenosine triphosphate (ATP) for rapid proliferation and migration, fatty-acid metabolism is particularly active. Healthy cells use diet-derived lipids preferentially, and their *de novo* synthesis of fatty acids is inhibited, so expression of the corresponding nascent lipid-metabolism enzymes is very low. However, the lipids of tumor cells are almost completely dependent on the lipid-neogenesis pathway, and are not regulated by the negative feedback of exogenous lipids. Phospholipids are key components of cell membranes. They are necessary raw materials for cell proliferation. Cell membranes contain phospholipids and cholesterol, which participate in various signal-transduction pathways of cell-membrane receptors. Active lipid neogenesis plays a key part in the growth and invasion of tumor cells^6^, ^7^.

Recently, increasing numbers of studies have demonstrated that lipid metabolism is also related to tumor drug-resistance. Liu and colleagues found that fatty acid synthase (FASN) can activate lipid metabolism in tumor cells to produce excess amounts of palmitic acid, thereby inhibiting the apoptosis of tumor cells and promoting multidrug resistance^8^. Giró-Perafita and coworkers demonstrated that inhibiting lipid synthesis in breast cancer cells could promote sensitivity to chemotherapy^9^. Yao and collaborators found that sorafenib-resistant tumors were accompanied by abnormal activity of the metabolism of glucose and lipids^10^. Inhibiting the metabolism of glucose and lipids in tumors could help to reverse sorafenib resistance.

Lipid metabolism is regulated directly by key enzymes such as ATP citrate lyase (ACLY), acetyl CoA carboxylase (ACC), FASN, and stearoyl COA desaturase 1 (SCD1)^11^-^14^. Several scholars have explored the relationship between the enzymes involved in lipid metabolism and sorafenib resistance. Those studies found that regulating the expression of SCD1, FASN and other enzymes involved in lipid metabolism can increase sensitivity to sorafenib. Bort and coworkers demonstrated that sorafenib-resistant hepatoma cells had active lipid metabolism. Further analyses of lipid-metabolism enzymes in sorafenib-resistant hepatoma cells showed that ACLY, ACC and FASN were activated to varying degrees, especially ACLY expression, which suggested that ACLY may play an important part in the sorafenib resistance of HCC^15^.

ACLY is a key enzyme in the first step of *de novo* lipogenesis. It is the most crucial enzyme for connecting glucose catabolism with anabolism of cholesterol and fatty acids. ACLY activity directly affects the activity of downstream lipid synthesis. ACLY can catalyze the conversion of citrate to acetyl coenzyme A, which is a key molecule in the intracellular synthesis of endogenous lipids (e.g., fatty acids, cholesterol)^16^. It has been demonstrated that ACLY has abnormal expression in various tumors (e.g., of the lung, breast, stomach, and colon), which is related to the prognosis of cancer patients^17^. Targeted inhibition of ACLY can help to arrest the proliferation of tumor cells^18^, ^19^. Recently, Wei and colleagues revealed the structure of ACLY, which is an important therapeutic target for cancer and metabolic disorders^20^. This discovery greatly enhanced the drug-development potential for ACLY for the treatment of cancer and metabolic disorders.

However, studies on the regulation of ACLY to change the resistance of sorafenib in HCC cells are lacking, and clinical studies on the relationship between ACLY expression and sorafenib resistance in HCC are sparse. Here, we undertook clinical and cellular experiments to investigate: (i) the correlation between ACLY expression and sorafenib resistance in HCC; and (ii) if targeted inhibition can reverse sorafenib resistance.

## Materials and Methods

### Ethical approval of the study protocol

The study protocol was approved by the Ethics Committee of the First Affiliated Hospital of Bengbu Medical College (Bengbu, China). Study procedures were in accordance with the Declaration of Helsinki (1975) and its later amendments. Written informed consent was obtained from all study participants for use of their resected tissue. Animal studies were conducted after approval from the Experimental Animal Committee of the First Affiliated Hospital of Bengbu Medical College.

### Participants

This was a retrospective study of patients confirmed to have HCC based on histopathology, and who underwent surgery.

Inclusion criteria were: (i) not in receipt of chemotherapy/radiotherapy before PET/CT; (ii) complete case records were available; (iii) tissue specimens were available for IHC staining.

Exclusion criteria were patients: (i) who could not comply with the study protocol; (ii) who had participated (or were participating) in other clinical trials; (iii) who could not complete imaging examinations (e.g., PET, CT) for specific reasons (e.g., claustrophobia, radiation phobia); or (iv) with severe liver and kidney insufficiency. Fifty-two samples of HCC were harvested from patients who underwent radical resection in the First Affiliated Hospital of Bengbu Medical College between January 2016 and June 2019. Tumor differentiation was graded according to the Edmondson-Steiner system. Patients were followed up in outpatient clinics. The level of alpha fetoprotein was measured. B-mode ultrasound of the liver and biliary tract was carried out every 3 months. Computed tomography (CT) was carried out in some patients.

### Positron emission tomography (PET)/CT

A combined PET/CT device (Biograph mCT; Siemens, Munich, Germany) was used for all PET/CT imaging. PET was carried out with an acquisition time of 3 min per bed position after CT. Patients received ^11^C-acetate (10 MBq/kg, i.v.). The mean uptake time was 20 ± 5 min. PET images were reconstructed iteratively, and CT data were used for attenuation correction. For quantitative analyses, two physicians with expertise in nuclear medicine evaluated ^11^C-acetate uptake on a workstation (MedEx, Beijing, China) by calculating the maximum standard unit value (SUV_max_): SUV_max_ = maximum pixel value in decay-corrected ROI activity (MBq/kg)/[radioactivity of the injected dose (MBq)/body weight (kg)], where ROI is the region of interest.

### Immunohistochemical (IHC) staining

IHC analyses were carried out on paraffin-embedded HCC tissues. After microtome sectioning to a thickness of 4 µm, slides were processed for staining. Primary antibodies against ACLY were purchased from Abcam (Cambridge, UK). Expression of each marker protein was measured according to a protocol reported previously. Slides were scored for staining intensity (0 to 3) and the percentage of cells with a score of “0” (0%), “1” (1% to 9%), “2” (10% to 49%), and “3” (50% to 100%) was determined. The IHC score (0 to 9) was defined as the product of the intensity and percentage of cells. Protein expression was judged to be “positive” if the IHC score was ≥4.

### Cell culture

Human HCC cell lines (HepG2, Huh7) were obtained from the Shanghai Institute of Cell Biology within the Chinese Academy of Sciences (Shanghai, China). HepG2 and Huh7 cells were maintained in Dulbecco's modified Eagle's medium (DMEM) supplemented with 10% fetal bovine serum, and 50 g/mL penicillin/streptomycin at 37°C under an atmosphere of 2% CO_2_ and 20% O_2_. Hypoxia was created with an atmosphere of 1% O_2_, 5% CO_2_, and 94% N_2_.

We wished to induce and establish drug-resistant cell lines. Hence, parental HCC cells were cultured in medium containing sorafenib. We used 0.5 μmol/L as the initial sorafenib concentration. When the cells could grow steadily, we increased the concentration by 0.5 μmol/L each time, until the target concentration of sorafenib (10 μmol/L) was reached. Flow cytometry was employed to detect apoptosis and verify the resistance of cells to sorafenib. The corresponding drug-resistant cell lines established by induction were named “HepG2-S” and “Huh7-S”.

### Western blotting

Forty-eight hours after transfection, cells were collected, and washed with phosphate-buffered saline (PBS). Total proteins were extracted from cell lysates and fractionated using sodium dodecyl sulfate-polyacrylamide gel electrophoresis. Then, the proteins were transferred onto nitrocellulose membranes for western blotting. Next, primary antibodies (anti-ACLY rabbit, 1:1000 dilution, Abcam; anti-β-actin, 1:2000, Sigma-Aldrich, Saint Louis, MO, USA) were used. Nitrocellulose membranes were washed extensively with PBS and incubated with secondary anti-rabbit antibody (1:10,000 dilution; LI-COR Biosciences, Lincoln, NE, USA).

### Glucose metabolism assay

Cellular uptake of ^14^C-glucose can be used to evaluate the glucose-uptake capacity of tumor cells. Cells were cultured in 12-well plates, then detached, and washed twice. Subsequently, they were incubated in 500 µL of DMEM containing 2 µCi/mL of ^14^C-glucose for 1 h at 37°C. Then, the cells were washed twice with ice-cold PBS. Cell lysates were produced using 500 µL of 0.1 M NaOH. Then, the radioactivity of the whole-cell lysates was assayed using a scintillation counter (LS 6500; Beckman Coulter, Fullerton, CA, USA). These readouts were normalized to the corresponding protein amounts (Beyotime Institute of Biotechnology, Beijing, China). Experiments were carried out independently in triplicate.

For ^14^CO_2_-release assay, cells cultured in 100-mm dishes were placed in an airtight chamber. Five milliliters of DMEM containing ^14^C-glucose (2 μCi/mL) were added and the cells were incubated for 2 h at 37°C with constant airflow (5% CO_2_ and 95% air, 15 mL/min); ^14^CO_2_ was trapped using 16 mL of an amine-based absorber. Four milliliters of absorber-trapped ^14^CO_2_ were transferred to vials containing 12 mL of Permafluor^®^ E+ (Perkin Elmer, Waltham, MA, USA) and radioactivity was counted using the liquid scintillation counter. The cell residue was used to determine protein content.

### Lipid synthesis assay

Samples were assayed for lipid synthesis through ^14^C-glucose. To begin the assay, 4 µCi/mL ^14^C-glucose were added to cells, which were incubated at 37°C for 6 h. Lipids were extracted from adherent cells grown on a 12-well plate by addition of 500 µL of a hexane:isopropanol solution. The wells were washed with an additional 500 µL of hexane:isopropanol solution. The extracts were combined and dried under N_2_, resuspended in 50 µL of chloroform, and the radioactivity was counted in a ScintiVerse BD Cocktail (Fisher Scientific, Pittsburgh, PA, USA) using the scintillation counter. Protein concentration was determined using a Bicinchoninic Acid Assay kit.

### Disulfide-crosslinked polyethylenimine (SS-PEI)-mediated plasmid transfection

HepG2 cells (10^5^) were seeded in each well of a 12-well plate. A mixture of SS-PEI and pcDNA3.1-GFP was added, followed by incubation for 4 h at 37°C, and then the medium was discarded. Green fluorescent protein (GFP) expression per well was determined in triplicate under an inverted-phase contrast fluorescence microscope. The proportion of cells with GFP expression was counted by fluorescence-activated cell sorting. The excitation wavelength was 488 nm and detection (emission) wavelength was 520 nm.

### Model using nude mice

Female BALB/c nude mice (4-weeks-old) were obtained from Shanghai SLAC Laboratory Animals (Shanghai, China). Mice were inoculated subcutaneously (s.c.) with HepG2-S cells (5×10^6^) in serum-free medium. Mice were randomized into four groups of five: (i) control (SS-PEI/pshRNA-NC); (ii) sorafenib alone; (iii) SS-PEI/pshRNA-ACLY alone; and (iv) sorafenib combined with SS-PEI/pshRNA-ACLY. Three weeks after tumors were observed, the complex of SS-PEI and plasmid (30 µg of plasmid and SS-PEI at a 1:15 ratio) was injected *via* the central vein in the tail every 3 days on six occasions. Mice were then killed.

### Statistical analyses

Data are the mean ± SD and were analyzed using SPSS software v16.0 (IBM, Armonk, NY, USA). Correlations between ACLY expression and clinicopathological characteristics were evaluated using Student's *t*-test and Pearson's chi-squared (χ^2^) test. Overall survival was calculated using the Kaplan-Meier method, and comparisons were made using the log-rank test. All statistical tests were two-sided. P < 0.05 was considered significant.

## Results

### Relationship between ACLY expression and clinicopathologic features and prognosis of HCC patients

**Figure 1:**
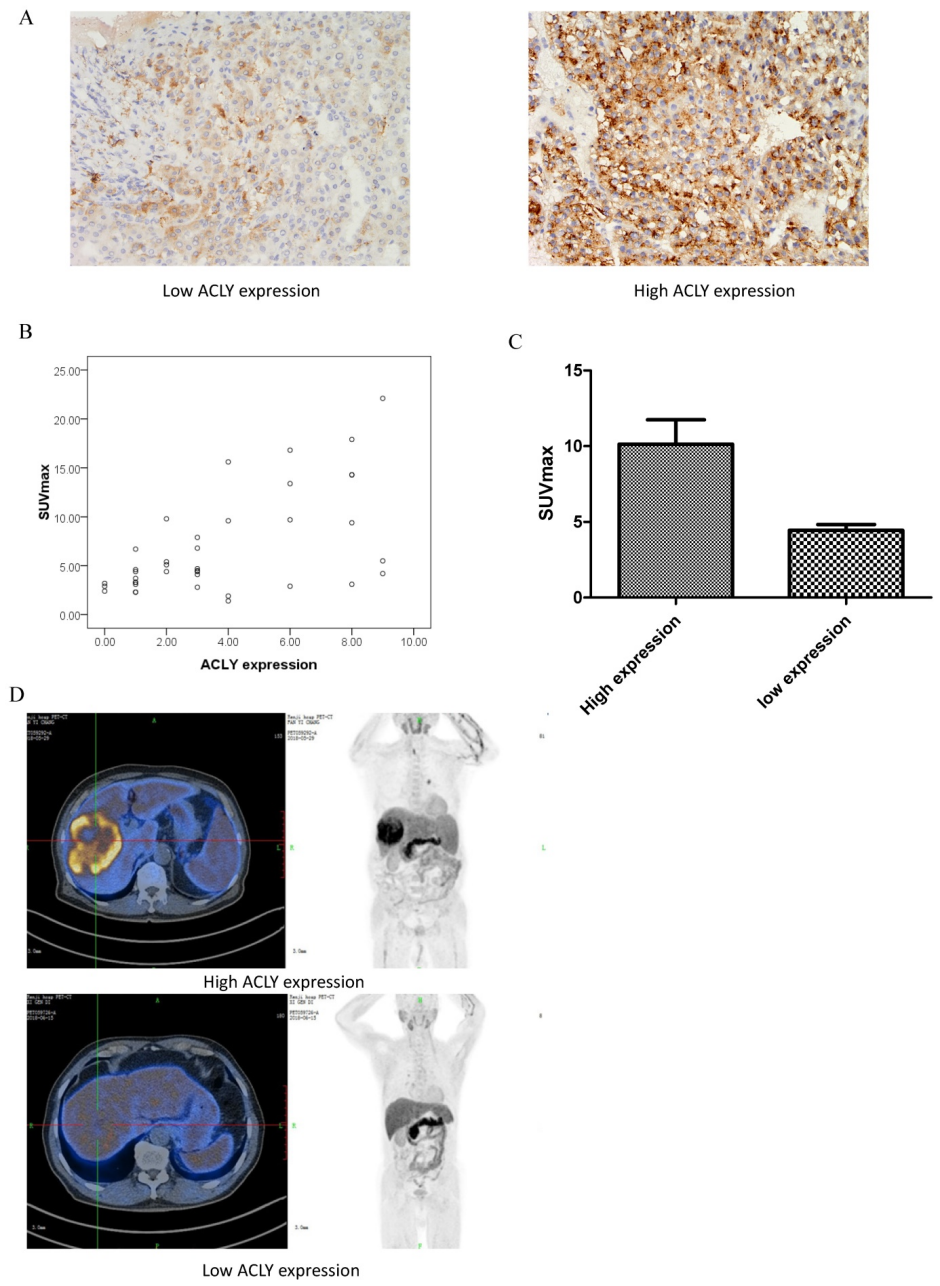
Correlation between ^11^C-acetate accumulation and ACLY expression. (A) ACLY expression in representative tumor tissues (×400 magnification). (B) SUV_max_ correlated inversely with the ACLY score (r = 0.48, P < 0.01). (C) Correlation between ^11^C-acetate accumulation and ACLY expression. SUV_max_ was significantly higher in tumors with high ACLY expression than in those with low ACLY expression (P < 0.01). (D) A 62-year-old man had hepatocellular carcinoma with high ACLY expression. ^11^C-acetate PET/CT showed intense accumulation of ^11^C-acetate in the tumor (SUV_max_ = 15.6) (top image). A 48-year-old man had hepatocellular carcinoma with low ACLY expression. ^11^C-acetate PET/CT showed moderate accumulation of ^11^C-acetate in the tumor (SUV_max_ = 3.2) (bottom image).

Lipid metabolism is closely related to the proliferation and invasion of tumor cells and drug resistance. ACLY is the first key enzyme in lipid synthesis in tumors. To assess the correlation between ACLY expression and clinicopathology in HCC, we retrospectively evaluated 52 patients with HCC, and measured ACLY expression (by IHC staining) in tumor tissue after biopsy (Figure 1A). In HCC tissue, ACLY expression was closely correlated (p < 0.01) with HCC differentiation. The greater the degree of HCC differentiation, the lower the ACLY expression. ACLY expression was significantly associated with portal-vein invasion and distant metastasis of HCC cells (P < 0.05). ACLY expression in patients with portal-vein invasion and distant metastasis was significantly lower than that in patients without portal-vein invasion (Table 1).

### ACLY expression in tumor tissues was closely related to lipid metabolism within the tumor

The lipid metabolism activity in tumor tissues can be reflected intuitively through PET/CT of tissues. Yoshimoto *et al.* suggested that an abnormal concentration of ^11^C-acetate in tumor tissue is related mainly to lipid synthesis within such tissue^21^. ^11^C-acetate can participate in the synthesis of free fatty acids. If tumor cells grow very fast, the lipid metabolism in cells shows abnormal activity. ^11^C-acetate can accumulate abnormally in tumor tissues that have more active lipid metabolism. Therefore, we retrospectively studied the correlation between ACLY in HCC and ^11^C-acetate metabolism. Of the 52 patients, 39 underwent PET/CT using ^11^C-acetate before treatment with sorafenib. The SUV_max_ of HCC lesions was closely related to ACLY expression (r = 0.48, P < 0.01) (Figure 1B). The SUV_max_ in the group with high expression of ACLY was significantly higher than that in the group with low expression of ACLY (Figure 1C, D). These observations reflected that ACLY expression in tumor tissues was closely related to lipid metabolism within the tumor.

### ACLY expression in HCC cells is related to sorafenib efficacy

Bort and colleagues demonstrated that ACLY expression in sorafenib-resistant HCC cells was significantly higher than sorafenib-sensitive cells 15. We wondered if ACLY expression in HCC patients was related to sorafenib resistance We divided HCC patients treated with sorafenib into an “ACLY low expression” group and “ACLY high expression” group. After sorafenib treatment, the relationship between ACLY expression and the therapeutic effect of sorafenib was compared. For evaluation of the efficacy of cancer treatment, we referred to the Response Evaluation Criteria in Solid Tumors (RECIST). Complete remission (CR) denoted that the lesions disappeared completely, no new lesions appeared, and this scenario was maintained for ≥4 weeks. Partial remission (PR) referred to the sum of the maximum diameter of lesions decreasing by ≥30% and this situation being maintained for ≥4 weeks. Stable disease (SD) denoted the sum of the maximum diameter of the lesions decreasing by <30% or increasing by <20%. Progressive disease (PD) was defined as the sum of the maximum diameter of lesions increasing by ≥20% and new lesions appearing. The effective rate was defined as: (CR cases + PR cases)/total cases × 100%. We found that sorafenib treatment was effective in 32.3% of patients in the group with low expression of ACLY, whereas it was only 14.3% effective in the group with high expression of ACLY (Table 2).

**Figure 2:**
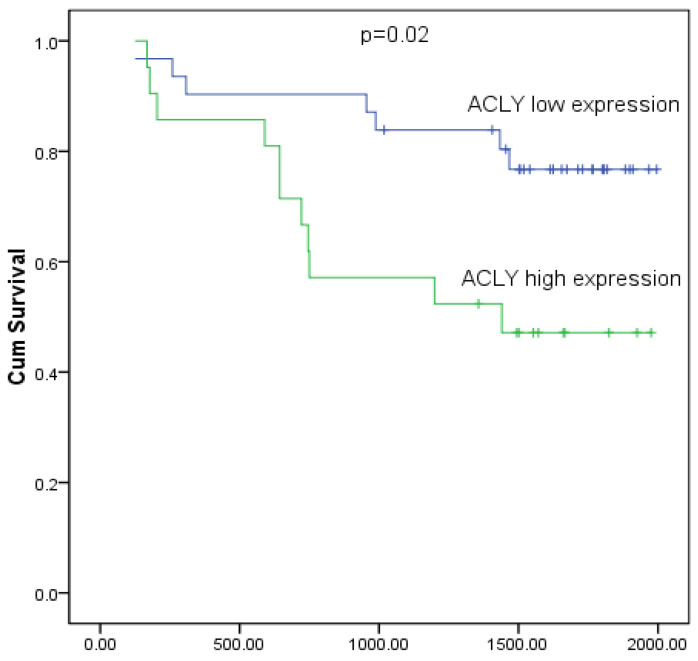
Kaplan-Meier analysis of survival of 52 patients with HCC stratified by ACLY expression. The log-rank test showed a significant difference between the groups (P = 0.02).

During a median follow-up of 1109 days for the 52 patients with HCC, there were 22 deaths (42.3%). The Kaplan-Meier survival curve showed that the survival of patients with high expression of ACLY was shorter than that of patients with low expression of ACLY (P < 0.05) (Figure 2).

### Lipid metabolism is more active in sorafenib-resistant HCC cells

**Figure 3:**
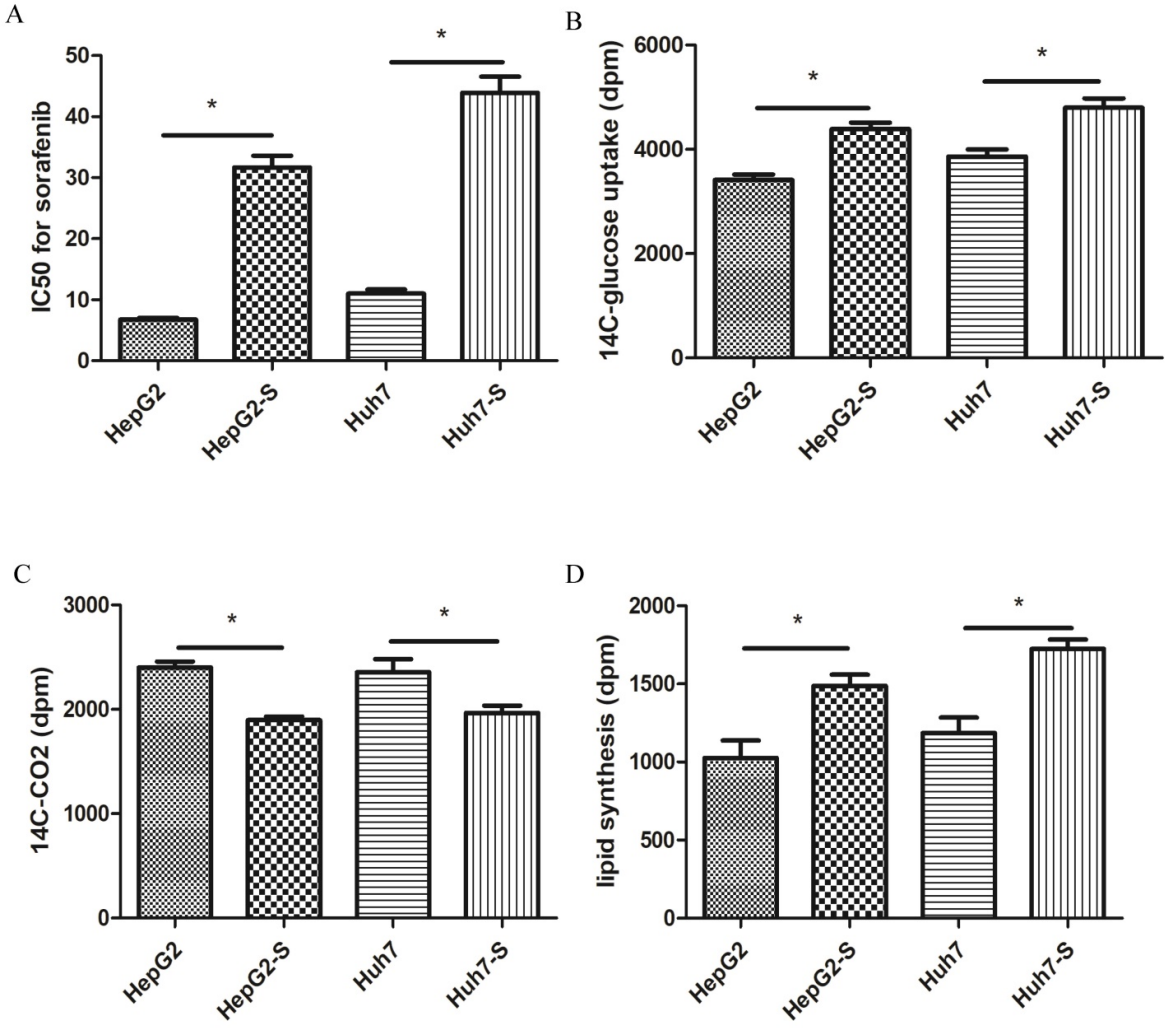
Characterization of alteration in the metabolism of glucose and lipids in HepG2 cells and sorafenib -resistant HepG2-S cells. (A) IC_50_ for sorafenib in HepG2 and HepG2-S cells. (B) ^14^C-glucose uptake by HepG2 and HepG2-S cells. (C) Release of ^14^C-CO_2_ by HepG2 and HepG2-S cells. (D) Comparison of lipid synthesis between HepG2 and HepG2-S cells. *P < 0.05. Data are the mean ± standard error of the mean (SEM) of three independent experiments.

In retrospective clinical studies, we found that HCC with high expression of ACLY was not sensitive to the treatment effect of sorafenib. We confirmed this result in HCC cells. The sorafenib-resistant cell lines that we created (HepG2-S, Huh7-S) and the corresponding parental cell lines (HepG2, Huh7) were cultured in medium containing 0, 5, 10, 15, 20, 25, or 50 μmol/L sorafenib for 48 h, respectively. The survival of HepG2 cells and HepG2-S cells was calculated. The half-maximal inhibitory concentration (IC_50_) of sorafenib in HepG2 cells was 6.80 μmol/L. The IC_50_ of sorafenib in HepG2-S cells was 30.58 μmol/L. Thus, HepG2-S cells were more resistant to sorafenib than HepG2 cells, with a resistance index of 4.50. The IC_50_ of sorafenib in Huh7 cells was 12.50 μmol/L, while the IC_50_ of sorafenib in Huh7-S cells was 43.80 μmol/L (Figure 3A). Therefore, Huh7-S cells had a resistance index of 3.50.

Furthermore, we analyzed the differences between the cells in glucose and lipid metabolism. First, we evaluated the glucose uptake and oxidative phosphorylation of these HCC cells. Compared with the parental cells, the uptake of ^14^C-glucose was higher in drug-resistant cells, but the ability to convert glucose to ^14^C-CO_2_ was lower (Figure 3B, C). Hence, the glycolytic activity of the drug-resistant cells increased, whereas the ability to carry out oxidative phosphorylation decreased. The Warburg effect in tumor cells not only provides sufficient ATP for proliferation of tumor cells, it also fulfills the high requirements for biological macromolecules. To determine the changes in fatty-acid metabolism in hepatocytes with sorafenib resistance, we used ^14^C-glucose as a carbon source to observe changes in the synthesis of lipids. Compared with HepG2 cells and Huh7 cells, the synthesis of lipids labeled with ^14^C in HepG2-S cells and Huh7-S cells increased significantly (P < 0.05) (Figure 3D). These results suggested that glucose and lipid metabolism were active, but that oxidative phosphorylation was decreased, in these two cell types. Hence, the increased amount of glucose taken up by cells did not undergo oxidative phosphorylation within the tricarboxylic acid cycle, but instead entered the lipid synthesis pathway. These actions led to increased metabolism of fatty acids, and more glucose was converted to fatty acids.

### ACLY expression is higher in sorafenib-resistant cells and the sensitivity of HCC cells to sorafenib increases after ACLY-knockout

Lipid metabolism is active in sorafenib-resistant cells, and ACLY is a key enzyme in lipid metabolism. We were very interested in ACLY expression in drug-resistant cells and the effect of changing ACLY expression on the resistance of tumor cells to sorafenib.

**Figure 4:**
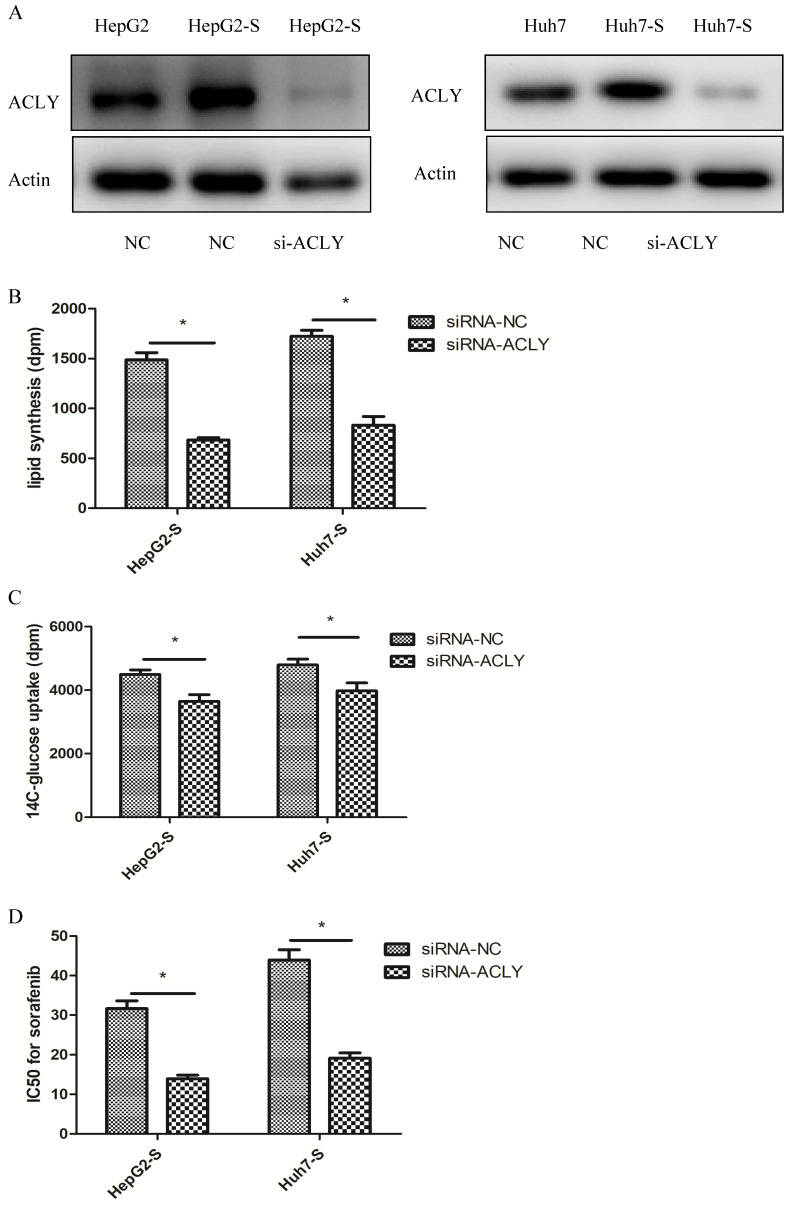
Inhibition of metabolism of glucose and lipids in HepG2-S cells by si-*ACLY*. (A) Expression of ACLY protein in HepG2, HepG2-S, and si-*ACLY* HepG2-S cells was detected by western blotting to evaluate the efficiency of *ACLY2*-knockdown 48-h post-transfection. (B) Comparison of lipid synthesis between si-*ACLY* HepG2-S cells and si-NC HepG2-S cells. (C) ^14^C-glucose uptake in si-*ACLY* HepG2-S cells and si-NC HepG2-S cells. (D) IC_50_ for sorafenib in si-*ACLY* HepG2-S cells and si-NC HepG2-S cells. *P < 0.05. Data are the mean ± standard error of the mean (SEM) of three independent experiments.

Western blotting revealed that ACLY expression in sorafenib-resistant (HepG2-S and Huh7-S) cells was increased significantly compared with that in HepG2 and Huh7 cells (Figure 4A). After ACLY-knockout, the ability of HepG2-S cells to synthesize fatty acids decreased by 53% (Figure 4B), glucose-uptake capacity (glucose metabolism) decreased by 19% (Figure 4C), and the resistance of HepG2-S cells to sorafenib decreased significantly (P < 0.01). Similarly, after knockout of ACLY expression, the ability of Huh7-S cells to synthesize fatty acids decreased by 48% (Figure 4B), glucose-uptake capacity (glucose metabolism) decreased by 17% (Figure 4C), and the resistance of Huh7-S cells to sorafenib decreased significantly (P < 0.01) (Figure 4D).

### ACLY-knockout reverses sorafenib resistance in HCC cells more significantly under hypoxic conditions

In hypoxic conditions, oncogenes such as hypoxia-inducible factor-1α of tumor cells are activated, which leads to active glycolysis and increased metabolism of fatty acids in tumor cells^22^. We were very interested in the effect of knocking out ACLY expression from HepG2 cells and Huh7 cells on inhibition of glucose metabolism, lipid metabolism, and sorafenib resistance under hypoxia.

**Figure 5:**
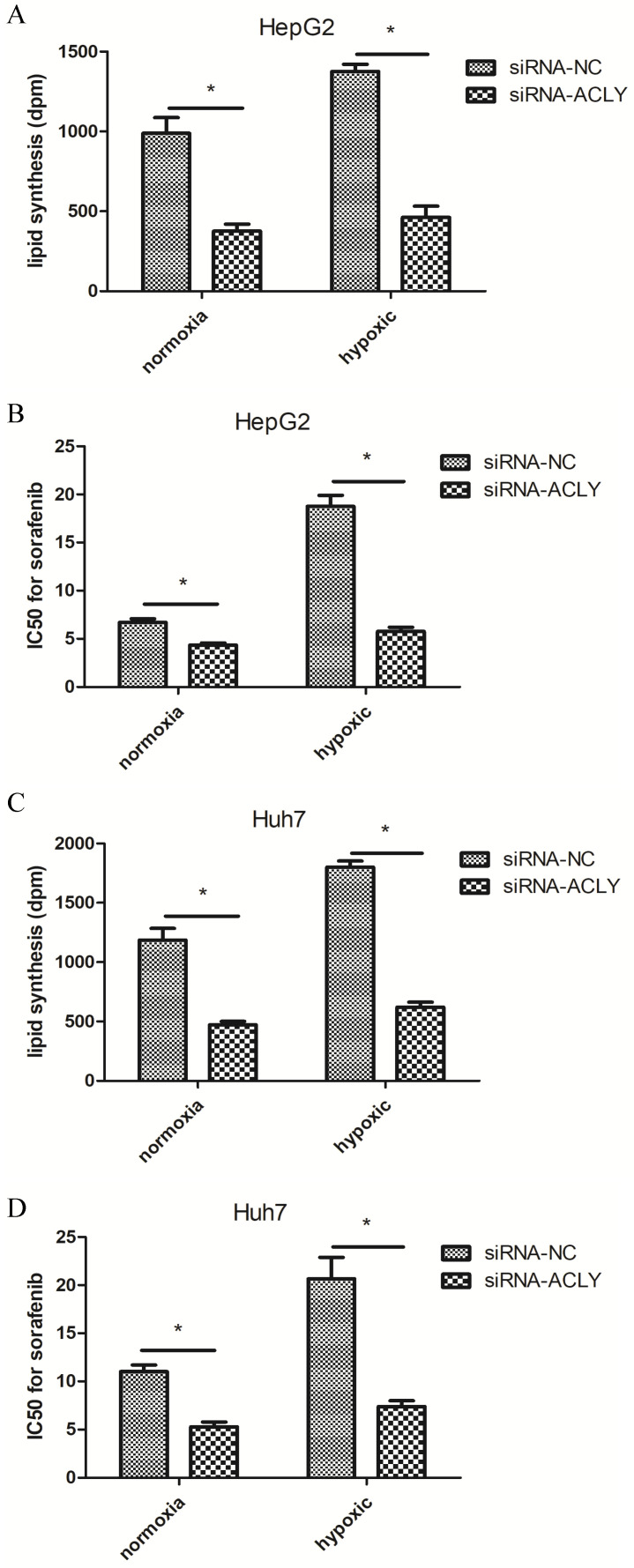
Effect of knockdown of ACLY expression on lipid synthesis and IC_50_ for sorafenib of HepG2 cells under hypoxia. (A) Compared with normoxia, knockdown of ACLY expression inhibited lipid synthesis more significantly in hypoxia. (B) ACLY-knockout increased sorafenib sensitivity in HepG2 cells more significantly under hypoxia.

Compared with the normoxic control group, under hypoxia (1% O_2_), the ability of HepG2 cells to produce ^14^C-labeled lipids increased significantly, and sorafenib resistance increased non-significantly (Figure 5A, B). In HepG2 cells and Huh7 cells under hypoxia, upon ACLY-knockout, not only did inhibition of fatty-acid synthesis become more significant, sensitivity to sorafenib increased, and the resistance reversal index of HepG2 cells and Huh7 cells reached 2.6 and 2.8, respectively. Therefore, compared with normoxia, hypoxia not only induced tumor cells to become more resistant to sorafenib but, in the case of high glucose metabolism, the recovery of drug sensitivity of tumor cells was also more significant after ACLY-knockout.

### SS-PEI/pshRNA-ACLY combined with sorafenib can inhibit the growth of drug-resistant cells significantly

**Figure 6:**
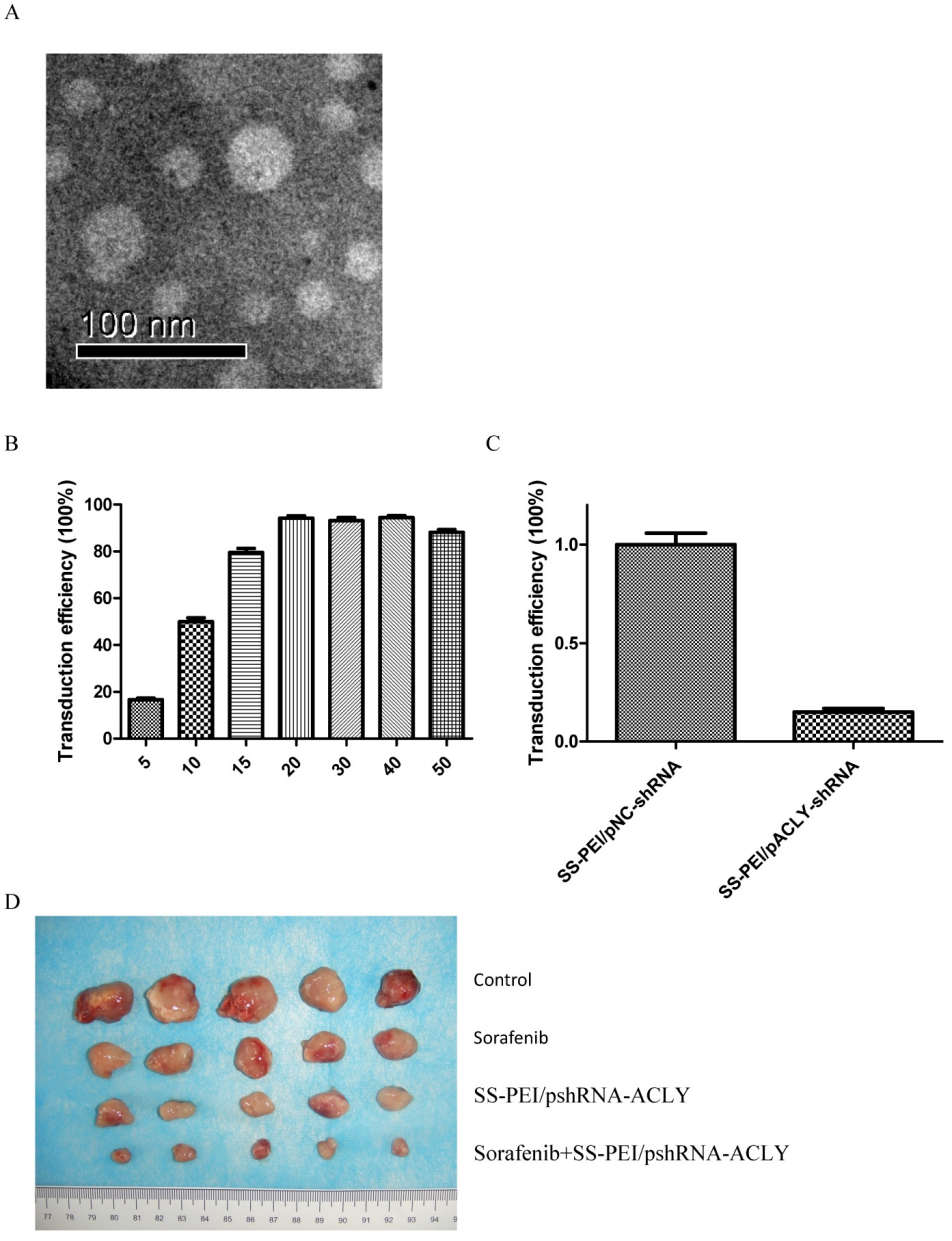

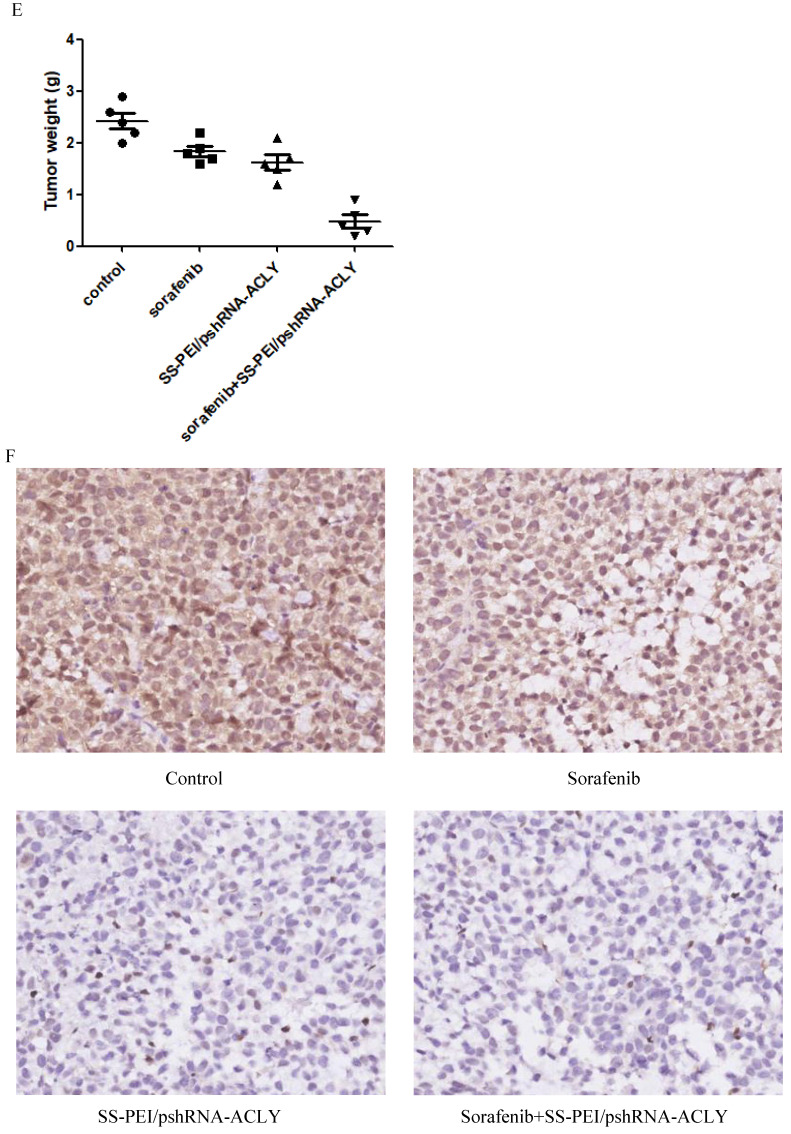
SS-PEI/pshRNA-ACLY combined with sorafenib treatment can inhibit the growth of drug-resistant cells significantly. (A) Electron micrograph of SS-PEI. (B) Transfection efficiency of complexes of SS-PEI/plasmid ACLY-shRNA-EGFP at different N/P ratios in HepG2 cells. (C) Effect of SS-PEI/pshRNA-ACLY-mediated silencing of the *ACLY* gene on mRNA expression. (D, E) *In vivo* growth of tumors in mice treated with sorafenib alone or in combination with SS-PEI/pshRNA-ACLY. *P < 0.05. (F) Representative immunohistochemistry images of ACLY from dissected tumors from different groups (200× magnification).

ACLY knockout reverses the sorafenib resistance of HCC. We used a vector that can carry the ShRNA-ACLY gene (by intravenous injection) to knockout the ACLY gene from tumor cells to kill the tumor or restore the sensitivity of the tumor to sorafenib *in vivo*. Disulfide-crosslinked polyethylenimine (SS-PEI) is a cationic polymer and a carrier of small interfering (si)RNA^23^. An electron micrograph of SS-PEI nanoparticles is shown as Figure 6A. We synthesized SS-PEI and used it to mediate plasmid transfection. *In vivo* in mice, we used SS-PEI as a transfection reagent to carry ACLY-interference plasmids. In this way, we inhibited ACLY expression in transplanted tumors to inhibit the growth of tumor cells and restore the drug sensitivity of drug-resistant cells.

First, we evaluated the transfection efficiency and knockout effect of SS-PEI/pshRNA-ACLY. We determined the mass ratio of SS-PEI and plasmids that achieved the highest transfection efficiency. For this, we constructed the expression plasmid pcDNA3.1-GFP with *GFP* and used SS-PEI-mediated pcDNA3.1-GFP to transfect HepG2 cells, then we measured intracellular GFP expression by flow cytometry. We achieved the best efficiency of transfection (96%) when the mass ratio of the plasmid and SS-PEI was 1:15 (15 μg/mL) (Figure 6B).

RT-PCR showed that SS-PEI/pACLY-shRNA transfection decreased the mRNA expression of ACLY significantly, by 82% (Figure 6C). Drug-resistant (HepG2-S) tumor-bearing mice were divided into four groups of five: i) control (SS-PEI/pshRNA-NC); (ii) sorafenib alone; (iii) SS-PEI/pshRNA-ACLY alone; and (iv) sorafenib combined with SS-PEI/pshRNA-ACLY. SS-PEI carrying SS-PEI/pshRNA-ACLY was injected into the tail vein once every 3 days. The mice were killed after six consecutive injections. The sorafenib+ACLY group showed significantly inhibited tumor growth compared with that in the sorafenib-alone group (Figure 6D, E).

We measured changes in the expression of ACLY protein in the tumor tissues of the four groups by IHC. ACLY expression in the SS-PEI/pshRNA-ACLY group was reduced significantly compared with that in the control group (Figure 6F).

## Discussion

Sorafenib has dual antitumor effects: (i) stopping proliferation of tumor cells by inhibiting expression of serine/threonine kinase in the Raf/MEK/ERK signaling pathway; and (ii) inhibiting tumor neovascularization. Sorafenib has a significant effect in treatment of advanced HCC, but resistance to its effects is a major problem^24^, ^25^. The drug-resistance mechanism of sorafenib is not clear. Also, there is no molecular marker to predict the therapeutic effect of sorafenib treatment. In addition, how HCC develops is not known.

Tumor metabolism is reflected in changes in signaling pathways within tumor cells. Our clinical data revealed that HCC cells had a high capacity for acetate intake and active lipid metabolism, which hampered sorafenib treatment and was likely to produce sorafenib resistance. Our data suggest that lipid metabolism in HCC is closely related to the sensitivity of HCC cells to sorafenib. ACLY is a key protein in lipid metabolism 26. We demonstrated, at the clinical and cellular levels, that ACLY is closely related to sorafenib resistance in HCC. At the clinical level, ACLY expression in HCC was related to the degree of tumor differentiation. ACLY expression in poorly differentiated HCC was significantly higher than that in moderately differentiated HCC, which showed that ACLY is closely related to the degree of malignancy of HCC. We undertook a retrospective analysis of the correlation between ACLY expression in HCC and the therapeutic effect of sorafenib; we found that 32.3% of patients in the group with low ACLY expression had efficacious sorafenib treatment, 14.3% of cases with high ACLY expression had efficacious therapy, and the difference between the two groups was significant. These data suggest that HCC with high ACLY expression is not sensitive to sorafenib therapy.

At the cellular level, we established a sorafenib-resistant model in HepG2 and Huh7 cells. We discovered that ACLY expression in sorafenib-resistant HCC cells increased significantly, as did the level of glucose metabolism and lipid metabolism, compared with sensitive cells. Similarly, after overexpressing the ACLY protein, the fatty-acid metabolism and glucose metabolism of HCC cells increased, and the resistance to sorafenib increased significantly. Conversely, when we knocked out ACLY expression in sorafenib-resistant HCC cells, the latter regained resistance to sorafenib. These results show that ACLY is closely related to the resistance of sorafenib in HCC. ACLY could not only be employed as a predictor of sorafenib resistance, but ACLY inhibition could promote the sensitivity of drug-resistant cells to sorafenib. More interestingly, compared with normoxia, inhibiting ACLY under hypoxic conditions could significantly inhibit fatty-acid synthesis in tumor cells and the Warburg effect, and the resistance of tumor cells to sorafenib was also reversed significantly. This result is very important because, in hypoxic conditions, tumor oncogenes are activated and antitumor therapy becomes more difficult: these problems could be overcome by ACLY-targeted therapy.

In this study, we used a type of “nanoreagent”, SS-PEI, to transfect plasmid pshRNA-ACLY. SS-PEI is a cationic polymer and, as a carrier of small interfering (si)RNA, it has high cellular infection efficiency 23, 27. In the present study, we assessed the efficacy of targeted therapy for HCC using SS-PEI as a gene vector through *in vitro* transfection or *in vivo* intravenous injection of plasmid carrying ACLY siRNA. Using SS-PEI-carrying plasmids for targeted gene therapy to inhibit ACLY expression proved efficacious. In a mouse model, after sorafenib-resistant HepG2-S cells had been transplanted into tumors, the tumor volume in the sorafenib group decreased only slightly, but the tumor volume in the group treated with sorafenib combined with SS-PEI/pshRNA-ACLY was suppressed significantly. During the treatment, the mice had a good mental state, and there was no significant change in body weight, liver function, or kidney function.

## Conclusions

ACLY is not only a key enzyme of lipid metabolism, it also an oncogene, and it is closely related to sorafenib resistance in HCC. Downregulation of ACLY expression can reverse sorafenib resistance effectively. *In vivo* and *in vitro* studies revealed the effectiveness and safety of using SS-PEI to mediate shRNA-ACLY transfection in the treatment of HCC, which could be promising targeted treatment for HCC and aid reversal of sorafenib resistance.

## Figures and Tables

**Table 1:** Relationship between ACLY expression and clinicopathologic features of HCC (n = 52)

Clinical variable	ACLY (IHC staining)		P
	Low expression	High expression	
Age (years)			
>60	19	10	0.33
<60	12	11	
Sex			
Male	23	15	0.97
Female	8	6	
Tumor differentiation			
I-II	20	4	<0.01
III-IV	11	17	
Vascular invasion			
Yes	11	14	0.03
No	20	7	
N staging			
Without lymphatic metastasis	18	8	0.16
With lymphatic metastasis	13	13	
M staging			
Without distant metastasis	22	5	0.01
With distant metastasis	9	16	

**Table 2:** Comparison of clinical efficacy between groups of patients

Group	CR	PR	SD	PD	Efficacy	*p*
ACLY low expression	0	10	13	8	32.26%	<0.01
ACLY high expression	0	3	7	11	14.29%	
